# Classification of Laser Vaccine Adjuvants

**DOI:** 10.4172/2157-7560.1000307

**Published:** 2016-02-12

**Authors:** Satoshi Kashiwagi, Timothy Brauns, Mark C Poznansky

**Affiliations:** Vaccine and Immunotherapy Center, Division of Infectious Diseases, Department of Medicine, Massachusetts General Hospital, 149 13th Street, Charlestown, Massachusetts, 02129, United States of America

**Keywords:** Laser, Ultra-short pulsed, Non-pulsed, Fractional, Conterminal, Adjuvant, Vaccine

## Abstract

An immunologic adjuvant, which enhances the magnitude and quality of immune responses to vaccine antigens, has become an essential part of modern vaccine practice. Chemicals and biologicals have been typically used for this purpose, but there are an increasing number of studies that are being conducted on the vaccine adjuvant effect of laser light on the skin. Currently, four different types or classes of laser devices have been shown to systemically enhance immune responses to intradermal vaccination: ultra-short pulsed lasers, non-pulsed lasers, non-ablative fractional lasers and ablative fractional lasers. Aside from involving the application of laser light to the skin in a manner that minimizes discomfort and damage, each type of laser vaccine adjuvant involves emission parameters, modes of action and immunologic adjuvant effects that are quite distinct from each other. This review provides a summary of the four major classes of “laser vaccine adjuvant” and clarifies and resolves their characteristics as immunologic adjuvants. These aspects of each adjuvant’s properties will ultimately help define which laser would be most efficacious in delivering a specific clinical benefit with a specific vaccine.

## Short Communication

An immunologic adjuvant is defined as a component that can accelerate, prolong, or enhance the quality of adaptive immune responses to an antigen [1–3]. Traditionally, adjuvants have been composed of one or more chemical and/or biological substances. A growing number of studies suggest that a physical parameter, namely laser light, should be considered as a new class of adjuvant. In this review, we will explore a classification for the different “laser vaccine adjuvants” and compare and contrast their distinct characteristics.

The increased use of vaccine antigens in the form of highly targeted recombinant molecules often requires an immunologic adjuvant to achieve desired immune effects [2,4,5]. A fundamental dilemma in adjuvant development is that highly effective adjuvants often result in unacceptable local reactogenicity or systemic toxicity [6–9]. As a result, only a limited number of adjuvants have made it into use with clinically approved vaccines [1,10,11]. New adjuvant approaches that combine sufficient efficacy with high degrees of safety are needed.

In recognition of the particular advantages of intradermal vaccination for targeting antigen-presenting cells [12–14] and sparing antigen dose [15–17], a growing range of technologies is now in use for intradermal delivery of vaccines [15,18]. Nevertheless, intradermal vaccines may still require adjuvanting to reduce the antigen dose or the number of vaccinations [19]. However, development of adjuvants is particularly challenging for intradermal vaccines. Few conventional adjuvants are likely to be appropriate for use in the skin due to issues related to viscosity, formulation with antigen, or the potential to induce persistent inflammatory signaling [20] that can cause unacceptable reactogenicity [21]. Therefore, a new class of safe and effective adjuvants for intradermal vaccination would constitute a major advance in the field of vaccination.

Against the background of a paucity of appropriate chemical and biological adjuvants for intradermal vaccines, a growing number of reports have shown that cutaneous lasers could play a useful role as immunologic adjuvants for intradermal vaccines. Since laser is a physical parameter, it does not persist in exposed tissues and is much less likely to induce tissue inflammatory responses in comparison to either chemical or biological adjuvants. This characteristic greatly favors its intradermal application and use for immunologic adjuvants for intradermal vaccines. To date, there are four major classes of “laser vaccine adjuvant” that fall into two broad categories based on the nature of the laser treatment of the skin (Table 1).

## Conterminal Laser Adjuvants

The first category of laser vaccine adjuvants utilizes laser beams that cover the entire target tissue and generate microscopic or cellular-level effects uniformly across this exposed tissue. The laser treatment characteristic of conterminal laser adjuvants typically lasts for many seconds or minutes and results in changes in cell signaling and tissue microarchitecture that enhance antigen presenting cell activation and trafficking. There are two classes within this category: ultra-short pulsed and non-pulsed laser vaccine adjuvants distinguished by the manner in which the light dose is delivered.

### Ultra-short pulsed laser vaccine adjuvants

Ultra-short pulsed laser vaccine adjuvants (UPLVAs) consists of laser pulses with durations in the nanosecond range or shorter to induce microscopic alterations to cellular or tissue functions that enhance immune responses. These ultra-short pulses may be delivered at high repetition rates into the thousands of Hertz and treatment may last over several minutes. The initial effects of absorbed laser photons from ultra-short pulses occur at an extremely small scale, generating significant photothermal and photoacoustic stress within the tissue or creating permeabilization of cell or organelle membranes. No frank tissue damage results from such treatment.

UPLVA was first described by Russian scientists utilizing copper vapor lasers that emit nanosecond pulses of yellow 510 nm and green 578 nm light with kilohertz repetition rates. A series of studies exploring laser enhancement of prophylactic and therapeutic vaccines were conducted in mice and humans by Onikienko et al. [22,23]. These studies used a copper vapor laser emitting a mix of 10% 510 nm and 90% 578 nm light emitted in 10–25 nanoseconds pulses at repetition rates of 5–20 kHz. Average irradiances (power density) used were between 1–6 W/cm^2^ on circular skin exposures of about 5–10 mm in diameter. The laser treatments typically lasted between 1–3 minutes followed immediately by intradermal vaccination. Their approach was tested with commercial prophylactic influenza and hepatitis B vaccines and resulted in significant increases of antigen-specific antibody titers in mice and in human volunteers previously documented as being non-responders to the influenza vaccine being tested. These investigators also explored the potentiating effects of copper vapor lasers with experimental therapeutic vaccines for chronic hepatitis B and cancer in human subjects [23]. Amongst all the laser vaccine adjuvants, UPLVA is the only clinically tested vaccine adjuvant.

Onikienko et al. characterized these laser exposures as being non-damaging to skin tissue by macroscopic and histological examinations and identified extracellular heat shock protein 70 (HSP70) as being an important mediator of the laser-induced vaccine response [22]. They concluded that the laser treatment induced rapid release of HSP70 by skin fibroblasts and/or keratinocytes and posited that this release enhanced immune responses via recruitment and activation of Langerhans cells.

Studies on UPLVAs have been continued in the U.S. over the last decade. Chen et al. conducted a series of studies with a Q-switched neodymium-doped yttrium aluminum garnet (Q-Nd:YAG) laser emitting 532 nm light in 6–7 nanosecond pulses at a repetition rate of 10 Hz. Irradiances used were 0.78 W/cm^2^ and skin exposures were 7 mm spots. This team found that treatment of mouse skin for 2 minutes increased humoral immune responses to a model vaccine [24], an inactivated influenza vaccine [24], a nicotine vaccine [21] and a dendritic cell cancer vaccine [25]. Chen et al. also showed that the Q-Nd:YAG laser increased motility and migration of MHC class II-positive APCs after laser treatment and enhanced antigen presentation. In addition, although they did not detect any tissue destruction or inflammation on routine histological examination, they observed that the green light pulsed laser treatment, featuring a pulse peak power over 2,500 times greater than that used by the Russians, resulted in derangement of the extracellular matrix in the dermis, which they postulated enhanced migration of antigen presenting cells (APCs) in the skin [24].

More recently, Kashiwagi et al. explored the use of a Q-switched neodymium-doped yttrium orthovanadate (Q-Nd:YVO4) laser, emitting either 532 nm or 1064 nm light in 7 nanosecond pulses at a repetition rate of 10 kHz, to adjuvant influenza vaccine. The pulsed 532 nm laser most closely matched the parameters of the Russian laser, with pulse peak powers (~2,800 kW), average irradiances (1 W/cm^2^), skin exposure targets (5 mm diameter spots) and total laser doses that were roughly equivalent to the copper vapor laser parameters. For the 1064 nm laser, the irradiance was 5 W/cm^2^ on 5 mm spots. The use of higher irradiances than typically used by Onikienko et al. or Chen et al. was related to the lower optical absorption coefficient of the skin at this wavelength. Up to that point only visible light (yellow and green) lasers had been studied as vaccine adjuvants, but Kashiwagi and his colleagues demonstrated that near-infrared UPLVAs could also induce enhancement of vaccine titers to a model vaccine [26].

At pulse durations in the low nanosecond range and shorter, thermal confinement of the absorbed laser pulse or, at higher intensities, nonlinear optical phenomena, can result in microcavitation within the exposed tissue, which can damage or destroy cells or, at lower pulse energies, lead to permeabilization of the cell membranes or organelles [27–30]. An Israeli team [31] has explored using the cell permeabilizing effects of a femtosecond pulsed lasers to enhance the uptake of DNA vaccines in the skin of mice.

For animal vaccination studies they used a titanium-sapphire, mode-locked laser (780 nm) emitting 200 femtosecond pulses with a 76 MHz repetition rate operating at 30 mW. The laser had a focal diameter of only 0.5 μm and a lens was used to focus the laser at depth of about 400 μm. The laser was moved around to create a ring of exposure over a two-minute period. The investigators administered a DNA plasmid of the hepatitis B surface antigen (HBsAg) intradermally followed 30 seconds later by laser treatment.

The laser treatment increased transfection efficiency of the plasmid and resulted in increased immunogenicity of the vaccine, with higher antibody titers of both IgG1 and comparable IgG2 compared to DNA vaccination alone, a 6-fold increase in cell proliferative responses to HBsAg epitopes, and increased release of IFN-γ by stimulated cells. In a viral challenge study, mice receiving the laser-enhanced DNA vaccine had lower levels of viral replication and circulating viral DNA compared to those receiving DNA vaccine alone.

While the femtosecond laser facilitates delivery of DNA into the cell, presumably through its membrane permeabilization effects, it enhances trafficking of the DNA to the cellular replication site and improves both TH1 and TH2 responses. From this perspective it can be considered an adjuvant for DNA vaccines.

Collectively, the class of ultra-short pulsed laser vaccine adjuvants concentrates the effects of photon absorption in tissues at a sub-cellular scale, producing cellular and tissue stress, microscopic tissue damage, or poration of cellular or organelle membranes that can enhance vaccine responses. These effects are created without inducing pain or causing obvious tissue damage.

A major concern for using laser light in the green or yellow spectrum (e.g., at 511, 532, and 578 nm) is that they are substantially absorbed by melanin, resulting in highly variable light absorption across different skin colors. The coefficient of absorption of melanin to near-infrared light is nearly 10-fold less than to green light, making NIR laser light much more uniformly absorbed by different skin colors. Amongst the ultra-short pulsed laser adjuvants, 1064 nm laser is therefore most feasible for clinical use.

### Non-pulsed laser vaccine adjuvant

While UPLVAs utilize ultra-short laser pulses to stimulate immune responses, a second class of conterminal laser adjuvants do not require laser pulsing at all. Non-pulsed laser adjuvants (NPLVAs) feature continuous wave laser light to stimulate immune responses. Currently, NPLVAs utilize the near infrared (700–1400 nm) region of the electromagnetic spectrum.

Discovery of the NPLVA approach originated from the Kashiwagi laboratory and was a result of comparative studies done among a continuous wave 1064 nm (1064CW) laser and 532 nm and 1064 nm UPLVAs. Surprisingly, one-minute exposures of the skin to the 1064CW laser provided superior antibody titers in a model vaccine study in mice, yielded a superior IgG response to an H1N1 vaccine, and conferred better protection in a lethal challenge study compared to UPLVAs [26]. This superiority is mediated by a mixed TH1–TH2 immune response to influenza vaccination induced by NIR laser treatment. In comparison, the UPLVA 532 nm laser augmented only TH1 response and failed to confer protection in this model.

Studies with the 1064CW laser used 5 W/cm^2^ irradiance and an exposure time of one minute on a 5 mm spot. Compared to the significant tissue stress or micro-tissue damage that may be induced by UPLVAs, NPLVAs do not result in any structural damage. Irradiances and total doses are limited to remain below the level of pain or tissue damage. Use of lasers in the near infrared spectrum largely avoids significant absorption by melanin or hemoglobin and allows for a larger dose to be delivered to the target tissue.

Kashiwagi et al. also demonstrated that the 1064CW laser treatment induces the expression of selected cytokines and chemokines in skin including CCL2 and CCL20, which ultimately induce functional and migrational changes in DCs in the skin, and subsequent migration and activation of CD11c+ dendritic cells. The mechanisms of action of NPLVAs appear to modify the immunologic environment in skin via photochemical reactions leading to the expression of selected cytokines and chemokines and APC activation and migration in skin.

### Fractional laser adjuvant

Fractional lasers utilize very small diameter beams to create controlled damage to a small fraction of the skin within the target area that generates wound-healing responses within surrounding untreated skin. The laser treatment characteristic of fractional laser adjuvants takes only a matter of seconds to create a regularly spaced array of vertical, submillimeter-diameter columns of damaged or ablated skin within the epidermis and dermis. Wavelengths and pulse power are selected that minimize damage outside the treated tissue, and the size, density and depth of the laser treatment zones are all carefully controlled to maximize the wound healing response while minimizing pain. Fractional laser devices are currently used in treating a variety of skin conditions including scarring and photodamage, as well as in skin rejuvenation treatments [32]. There are two classes of fractional laser adjuvants: non-ablative and ablative, distinguished by the type of controlled damage the laser microbeams cause to the skin.

### Non-ablative fractional laser adjuvant

Non-ablative fractional laser vaccine adjuvants (NAFLVA) utilize controlled coagulation of tissue to produce immune-enhancing effects in the skin. These lasers emit a series of microbeam pulses (typically in the range of 1410 to 1550 nm) to progressively coagulate a series of vertical columns of tissue, usually 50–150 μm in diameter, extending from near the surface of the epidermis into the dermis at a depth of about 400 μm. The depth of the column is controlled by the number of laser pulses used at a particular point. The resultant arrays of coagulated skin columns are on the scale of a few square millimeters and take only a matter of seconds to create. The width and density of these columns of coagulated tissues, called microthermal zones (MTZs), can be carefully controlled to induce sterile inflammatory responses in the skin that lead to rapid tissue regeneration at the site of damage.

Recently NAFL devices have been used to create an adjuvant effect for vaccination. Wang et al. used a commercial NAFL device where 10 millisecond pulses (pulse energy 15 mJ) of a 1410 nm laser microbeam at a repetition rate of 15.4 Hz generated an array of MTZ at a density of 54–93 MTZ/cm^2^ over a 7 × 10 mm rectangle. Treatment of the vaccine inoculation site was completed in a matter of seconds using two passes [33,34]. NAFL treatment followed by intradermal model or influenza vaccination augmented humoral immune responses, induced cross-protective immunity and conferred protection in a lethal challenge murine model of influenza [33,34]. Wang et al. demonstrated that dying cells in the MTZs release damage-associated molecular patterns (DAMPs) and dsDNA. DAMPs attract APCs, in particular, plasmacytoid dendritic cells (pDCs), leading to the stimulation of humoral responses [33] and dsDNA activate the dsDNA-sensing STING pathway to augment CD8+ T-cell responses, conferring cross protection [34].

### Ablative fractional laser adjuvant

Ablative fractional laser vaccine adjuvants (AFLVAs), utilize a similar approach to NAFLVAs to create microtreatment arrays in the skin. The wavelengths chosen 2790, 2940 or 10,000 nm results in high absorption by water and subsequent explosive superheating of the aqueous content of the tissue column. This leads to progressive ablation of the narrow columns of tissue rather than coagulation. In ablative fractional laser (AFL) treatment, an array of tiny holes (50–150 μm in diameter) is drilled into the surface of the skin, typically to a depth of less than one millimeter. Each microhole within the array is surrounded by a very narrow (about 5 μm) layer of coagulated tissue.

In addition to its range of clinical applications in skin repair and regeneration, AFL was investigated early on as a means of enhancing transcutaneous delivery of drugs [35] and, more recently, as a vaccine delivery approach. Since the skin is enriched with APCs, delivery of vaccine antigens to the skin is considered to be a more effective vaccination approach than intramuscular or subcutaneous delivery, and existing literature indicates that such use of lasers is safe and effective in enhancing the drug delivery through the skin [36]. The ablative fractional laser approach appears to not only enhance vaccine immunogenicity by enabling intradermal delivery of antigens to APCs, but also acts as an adjuvant by altering immunologic responses in the skin to vaccines [37].

Terhorst et al., used the P.L.E.A.S.E.^®^ (Precise Laser Epidermal System) device developed by Pantec Biosolutions AG (Ruggell, Liechtenstein), which is a 2940 nm diode-pumped, erbium:yttrium-aluminium-garnet (ER:YAG) laser releasing 75 μs pulses at a repetition rate of 200 Hz in studies of vaccine delivery. Skin exposures were 14 mm^2^ spots. The treatment of skin on this spot lasted only a few seconds and resulted in an array of several hundred micropores of approximately 150 μm diameters that quickly filled with exudate. While the goal of the study was to deliver a model antigen to dermal XCR1+ DCs through the laser-generated micropores in the epidermis, they observed significant increases in antigen-specific CD8+ and CD4+ T cell responses and anti-tumor efficacy of prophylactic and therapeutic cancer vaccines in the absence of intentionally added adjuvants [37]. The mechanisms of action of AFL adjuvant are considered to be similar to those of NAFLVAs; the mild inflammatory milieu created by death of keratinocytes by skin laser microporation itself induces T cell responses.

## Conclusion

The four classes of laser vaccine adjuvant have distinct characteristics as immunologic adjuvants. Conterminal laser adjuvants evoke no overt histological changes and mainly enhance humoral immune response, while fractional laser adjuvants cause predictable histological changes that augment both humoral and cell-mediated immune responses. It is difficult to directly compare the efficacy of each kind of laser vaccine adjuvant because of the variability of the models used in these studies. However, since adjuvants receive regulatory approval for clinical use only with specific vaccines, the key to optimizing the utility of laser adjuvants is to correctly match the specific laser adjuvant with the right vaccine. To do so requires a better understanding of mechanism of action of each adjuvant.

Currently, a number of approved and clinical-stage adjuvants exist, with each one having a different mechanism of action. It is important to understand the mechanism of action of an adjuvant to direct a tailored vaccine formulation toward the desired clinical benefit and minimize unwanted side effects [38]. Since the majority of vaccines are intended for prophylactic use and administration to healthy populations, the safety requirements for an approved adjuvant has are stringent [39]. Understanding the role of a specific adjuvant in stimulation of an immune response is critical to maximize vaccine responses while minimizing any risk of an adverse event. From this point of view, further basic research on the mechanisms of action of laser vaccine adjuvants is warranted.

The current approach of delivering vaccines intramuscularly-into tissue that is poorly populated by immune cells-often requires chemical adjuvants that are capable of forming persistent inflammatory foci to attract antigen-presenting cells into this tissue, contributing significantly to the pain and risk involved in vaccination. As a broad range of transdermal, intradermal and mucosal vaccination technologies mature and enter clinical use [15,18,40], these vaccines will require a new kind of adjuvant. A small number of new adjuvants have been proposed that are ideally suited to use in the skin environment [40,41], and the four classes of laser vaccine adjuvant join this list. Studies on these laser adjuvants show that such laser treatments are safe, tolerable and do not induce significant uncontrolled damage to the skin tissue or result in a breach of its barrier function. The laser adjuvants have a number of inherent advantages over current chemical and biological adjuvants that support their further development.

They do not persist in the tissues, which reduces the risk of side effects from the adjuvant itself.

They are separate from the antigen and present no requirement of formulation or risk of alteration to the vaccine antigen. Vaccine antigens can therefore be used without modification.

Long-term storage is not an issue for a laser device and there are no special low-temperature storage requirements that chemical or biological adjuvants typically have. This may further enhance development of thermostable vaccines.

As a new technology approach, the laser adjuvant has not yet benefited from advanced preclinical development. Along with the demonstration of the safety and efficacy through rigorous preclinical safety and toxicology studies, an expanded number of approved intradermal vaccines, an increase in the number of vaccine antigens available for intradermal development, and the development of laser devices appropriate for use as adjuvants will be the key steps for the laser adjuvant to be employed in both prophylactic and therapeutic vaccines for infectious diseases as well as cancer.

## Figures and Tables

**Table 1:** Here we review these four major classes and discuss their similarity and distinctiveness as vaccine adjuvants.

Category		Laser type	Wavelength	Target Spot Size	Pulse Duration	Pulse Frequency	Pulse Energy	Average Power	Exposure Duration	Putative Mechanisms of action	Tested in	References
Conterminal	Ultra-short pulsed (UPLVAs)	Copper vapor	511/578 nm	5–10 mm in diameter	10–25 ns	5 – 20 kHz	60 μJ	1 – 6 W/cm^2^	1–3 min	Extracellular release of HSP70 to enhance APC activation and antigen presentation	Mice, humans	[22,23]
		Q switched Nd:YAG	532 nm	7 mm in diameter	6–7 ns	10 Hz	30 mJ	0.78 W/cm^2^	1–4 min	Derangement of connective tissue facilitates migration of APCs	Mice	[21,24,25]
		Q switched Nd:YVO_4_	532 or 1064 nm	5 mm in diameter	7 ns	10 kHz	67 μJ	1 – 5 W/cm^2^	1–4 min	Photochemical, photothermal and photoacoustic stimulation of tissue leading to APCs activation and migration	Mice	[26]
		modelocked Ti:Al_2_O_3_	780 nm	0.5 μm	200 fs	76 MHz	n.d.	n.d.	2 min	Membrane permeabilization effects	Mice	[31]
	Nonpulsed (NPLVAs)	CW Nd:YVO_4_	1064 nm	5 mm in diameter	N/A	N/A	N/A	5 W/cm^2^	1–4 min	Photochemical reaction in skin tissue leading to immunostimulatory microenvironment for APCs	Mice	[26]
Fractional	Nonablative (NAFLVAs)	Fractional ER:Glass	1410 nm	7 x 10 mm	10 ms	15.4 Hz	15 mJ	n.d.	1–2 sec	Sterile inflammation caused by an array of MTZs in skin recruit and activate DCs	Mice, pigs	[33,34]
	Ablative (AFLVAs)	Fractional ER:YAG	2940 nm	14 mm^2^	75 μs	200 Hz	n.d.	n.d.	Several seconds	The mild inflammatory milieu created in the dermis by skin laser microporation itself activates APCs	Mice	[37]

Nd:YVO4: Neodymium-Doped Yttrium Orthovanadate; Nd:YAG: Neodymium-Doped Yttrium Aluminum Garnet; ER:YAG: Erbium:Yttrium-Aluminium-Garnet; CW: Continuous Wave; MTZ: Microthermal Zone; APC: Antigen Presenting Cell; DC: Dendritic Cell; n.d: Not Described.
